# Use and Cost of Skin Biopsy Procedures in the Medicare Part B Fee-for-Service Population, 2017 to 2020

**DOI:** 10.21203/rs.3.rs-2669252/v1

**Published:** 2023-03-14

**Authors:** Yiwen Li, Travis Blalock, Richard Duszak, Howa Yeung

**Affiliations:** Emory University School of Medicine; Emory University School of Medicine; University of Mississippi Medical Center; Emory University School of Medicine

## Abstract

The Center for Medicare and Medicaid Services noted skin biopsies have high expenditures and changed biopsy billing codes in 2018 to better align procedure type and associated billings. We examined associations between billing code updates and skin biopsy utilization and reimbursement across provider specialties. While dermatologists perform most skin biopsies, the proportion of skin biopsies performed by dermatologists has continuously decreased, but the proportion of skin biopsies performed by nonphysician clinicians has increased from 2017–2020. After the code update, the non-facility national payment amount decreased for first tangential biopsy but increased for first punch, first incisional, additional tangential, additional punch and additional incisional biopsy compared to the corresponding amount for first and additional biopsy before the code update. The allowable charges and Medicare payment per skin biopsy increased across provider specialties but has increased the most for primary care physicians from 2018–2020.

In 2016, The Center for Medicare and Medicaid Services screened for potentially misvalued high-expenditure procedures. Two preexisting skin biopsy Healthcare Common Procedure Coding System (HCPCS) codes for first and additional biopsies were surveyed for correct valuation. To better align procedure type and associated billings, six new codes were created [3] by the Current Procedural Terminology panel which were surveyed and presented for valuation at the Relative Value Scale Update Committee in 2017. The six new codes became effective on January 1st, 2019. We aimed to examine associations between billing code changes on procedure use and payments across provider specialties.

Utilization rates per 1,000 beneficiaries were calculated using Medicare Part B enrollment data. Biopsy volumes, Medicare allowable charges and payments were obtained from Medicare Part B Physician Supplier Procedure Summary Master Files [5], sorted by provider specialties. Analysis focused on dermatologists, non-physician clinicians (NPCs) and primary care physicians (PCPs) who had the highest skin biopsy volume. Prices were adjusted using the Personal Consumption Expenditures-Health Index as 2020 US dollars.

Total Medicare fee-for-service skin biopsies increased from 5.1 million in 2017 to 5.3 million in 2019 and decreased to 4.6 million in 2020 corresponding to utilization of 154.6, 162.2, and 143.6 procedures per 1,000 beneficiaries. From 2017 to 2020, the proportion of skin biopsies performed by dermatologists and PCPs decreased (76.0% to 71.6%, and 2.4 to 1.8%), but increased from 19.5 to 24.7% for NPCs. The proportions of first tangential, punch and incisional biopsies didn’t meaningfully differ between dermatologists and NPCs (63.7% vs 63.0%, 28.5% vs 28.0%, 5.7% vs 6.8%), but PCPs performed a higher proportion of first incisional biopsies (27.2%) (Table 1).

Compared to the non-facility national payment amount for first skin biopsy in 2018, the amount for first tangential, punch and incisional skin biopsy changed by −8.1%, +15.5% and +39.8% respectively from 2019–2020. Compared to the non-facility national payment amount for additional skin biopsy in 2018, the amount for additional tangential, punch and incisional skin biopsy changed by +58.7%, +81.3% and +114.4% respectively from 2019–2020 (Figure 1). Comparing provider specialties, allowable charges per skin biopsy increased by 2.3%, 1.5% and 11.1% for dermatologists, NPCs, and PCPs respectively from 2018–2020; and Medicare payment per skin biopsy increased by 4.0%, 3.3% and 17.7% for dermatologists, NPCs, and PCPs respectively from 2018–2020 (Table 1).

From 2017 to 2020, a shift occurred in skin biopsy practice patterns among different clinicians managing cutaneous conditions. The number of NPCs in dermatology practice continues to grow with a higher density of NPCs than dermatologists seen in rural counties, which can be attributed to the imbalance of patient demand and the shortage of dermatologists in underserved regions, the cost-effectiveness of hiring NPCs, and the expansion of NPCs’ scope of practice [1]. These changes may impact patient access and quality of care related to diagnosis requiring skin biopsies as studies suggested NPCs required more skin biopsies to diagnose skin malignancy as compared with dermatologists [2, 4]. Compared to the skin biopsies performed by dermatologists and NPCs, PCPs performed fewer overall skin biopsies but with a larger portion of first punch biopsies.

Our data pertained to skin biopsy procedures in Medicare Part B fee-for-service beneficiaries and may not be generalizable to other populations. We lack patient or provider level data to examine underlying reasons for skin biopsy utilization changes. HCPCS code updates may influence skin biopsy utilization and reimbursement pattern across provider specialties. Monitoring the changes over time is important on dermatological care access and outcomes.

## Figures and Tables

**Figure 1:**
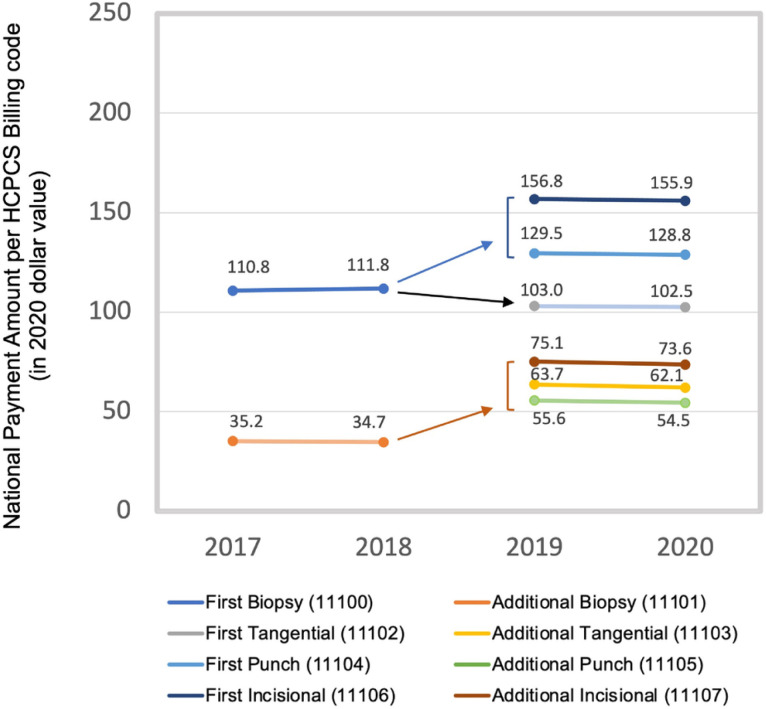
Medicare national payment amount by skin biopsy billing code 2017–2020. Medicare national payment amount for skin biopsies before and after the billing code update, 2017–2020. HCPCS, Healthcare Common Procedure Coding System. $2020, prices adjusted using the Personal Consumption Expenditures-Health Index as 2020 US dollars.

**Table 1.** Skin biopsy utilization, Medicare allowable charges and national payment amount 2017–2020.

Total skin-biopsies			2017	2018	2019	2020
			5,188,774	5,222,442	5,366,341	4,629,088
Medicare Part B Enrollment			33,562,359	33,366,109	33,077,390	32,245,820
Utilization per 1000 Medicare Part B fee-for-service beneficiaries			154.6	156.6	162.2	143.6
Allowable charges per skin biopsy (Adjusted to $2020)		Dermatologists	$77.2	$77.5	$79.5	$79.1
		NPCs	$65.4	$65.8	$67.1	$66.6
		PCPs	$76.7	$77.8	$85.7	$87.2
Medicare Payment per skin biopsy (Adjusted to $2020)		Dermatologists	$55.9	$56.4	$58.9	$58.4
		NPCs	$47.0	$47.3	$49.2	$48.5
		PCPs	$57.5	$58.5	$68.3	$69.4
**2017–2018**	First biopsy (11100)			Additional biopsy (11101)		
Dermatologists # of biopsies (% of all biopsies)	2743658 (70.0%)			1173781 (30.0%)		
NPCs # of biopsies (% of all biopsies)	744096 (69.9%)			319872 (30.1%)		
PCPs # of biopsies (% of all biopsies)	89400 (77.2%)			26420 (22.8%)		
**2019–2020**	First tangential (11102)	First punch (11103)	First incisional (11104)	Additional tangential (11105)	Additional punch (11106)	Additional incisional (11107)
Dermatologists # of biopsies (% of all biopsies)	230822 (63.5%)	1030515 (28.5%)	205990 (5.7%)	50902 (1.4%)	20039 (0.5%)	5163 (0.1%)
NPCs # of biopsies (% of all biopsies)	752087 (63.0%)	333949 (27.9%)	81168 (6.8%)	23601 (2.0%)	2493 (0.2%)	527 (0.04%)
PCPs # of biopsies (% of all biopsies)	42846 (46.7%)	18025 (19.6%)	24980 (27.2%)	3467 (3.8%)	2369 (2.6%)	123 (0.1%)

The table demonstrates skin biopsies performed among Medicare fee-for-service beneficiaries from 2017–2020 and Medicare allowable charges and payment per skin biopsy by dermatologists, non-physician clinicians, and primary care physicians. Non-physician clinicians, NPCs, includes physician assistants and nurse practitioners. Primary care physicians, PCPs, includes family practitioners, internal medicine providers and general practitioners. $2020, prices adjusted using the Personal Consumption Expenditures-Health Index as 2020 US dollars.

## References

[1] Coldiron B, Ratnarathorn M. Scope of physician procedures independently billed by mid-level providers in the office setting. JAMA Dermatol. Nov 2014;150(11):1153–9. doi:10.1001/jamadermatol.2014.1773 25110923

[2] Gronbeck C, Feng H. Volume and distribution of skin biopsies performed by dermatologists and other health care providers in the Medicare population in 2019. J Am Acad Dermatol. Nov 14 2021. doi:10.1016/j.jaad.2021.11.006 34785279

[3] Kircik LH. A closer look at the new biopsy codes. Cutis. 2019; 103(3):122–123. 31039228

[4] Patel S, Nguyen BT. Characterization of biopsies by dermatologists and nonphysician providers in the Medicare population: a rapidly changing landscape. Dermatol Surg. 2021;47: 1337–1341. 10.1097/dss.0000000000003150 34352835

[5] US Centers for Medicare & Medicaid Services. Medicare provider utilization and payment data: physician and other supplier public use file. https://www.cms.gov/Research-Statistics-Data-and-Systems/Statistics-Trends-and-Reports/Medicare-Provider-Charge-Data/Physician-and-Other-Supplier.html. Accessed 8 March, 2023.

